# Application of a high-throughput genotyping method for loci exclusion in non-consanguineous Australian pedigrees with autosomal recessive retinitis pigmentosa

**Published:** 2012-07-25

**Authors:** Rachel L. Paterson, John N. De Roach, Terri L. McLaren, Alex W. Hewitt, Ling Hoffmann, Tina M. Lamey

**Affiliations:** 1 Australian Inherited Retinal Disease Register & DNA Bank, Department of Medical Technology and Physics, Sir Charles Gairdner Hospital, Perth, Western Australia, Australia; 2 Lions Eye Institute, Centre for Ophthalmology and Visual Science, University of Western Australia, Perth, Western Australia, Australia

## Abstract

**Purpose:**

Retinitis pigmentosa (RP) is the most common form of inherited blindness, caused by progressive degeneration of photoreceptor cells in the retina, and affects approximately 1 in 3,000 people. Over the past decade, significant progress has been made in gene therapy for RP and related diseases, making genetic characterization increasingly important. Recently, high-throughput technologies have provided an option for reasonably fast, cost-effective genetic characterization of autosomal recessive RP (arRP). The current study used a single nucleotide polymorphism (SNP) genotyping method to exclude up to 28 possible disease-causing genes in 31 non-consanguineous Australian families affected by arRP.

**Methods:**

DNA samples were collected from 59 individuals affected with arRP and 74 unaffected family members from 31 Australian families. Five to six SNPs were genotyped for 28 genes known to cause arRP or the related disease Leber congenital amaurosis (LCA). Cosegregation analyses were used to exclude possible causative genes from each of the 31 families. Bidirectional sequencing was used to identify disease-causing mutations in prioritized genes that were not excluded with cosegregation analyses.

**Results:**

Two families were excluded from analysis due to identification of false paternity. An average of 28.9% of genes were excluded per family when only one affected individual was available, in contrast to an average of 71.4% or 89.8% of genes when either two, or three or more affected individuals were analyzed, respectively. A statistically significant relationship between the proportion of genes excluded and the number of affected individuals analyzed was identified using a multivariate regression model (p<0.0001). Subsequent DNA sequencing resulted in identification of the likely disease-causing gene as *CRB1* in one family (c.2548 G>A) and *USH2A* in two families (c.2276 G>T).

**Conclusions:**

This study has shown that SNP genotyping cosegregation analysis can be successfully used to refine and expedite the genetic characterization of arRP in a non-consanguineous population; however, this method is effective only when DNA samples are available from more than one affected individual.

## Introduction

Retinitis pigmentosa (RP) is the most common form of inherited blindness, with a total prevalence of approximately 1 in 3,000 to 1 in 4,000 people [1,2]. RP is an inherited retinal disease caused by degradation of rod and cone photoreceptor cells, leading to a progressive loss of vision. Inheritance of RP typically follows a Mendelian pattern, although rare digenic and mitochondrial forms also exist [3-5].

Recently, there has been significant progress in several gene therapies for RP and related diseases, in particular retinal pigment epithelium-specific 65 kDa protein (*RPE65*) gene therapy for treatment of Leber congenital amaurosis (LCA) in humans [6-11], two mouse models of aryl hydrocarbon receptor interacting protein-like 1 (*AIPL1*) gene therapy [12], and retinitis pigmentosa GTPase regulator (*RPGR*) gene therapy in canine models of X-linked RP [13]. For gene therapies such as these to be generally applicable, the disease-causing gene must be identified in each case. Therefore, genetic characterization is becoming increasingly important for identifying candidate individuals for gene therapy.

Autosomal recessive retinitis pigmentosa (arRP) is extremely genetically heterogeneous, with more than 35 causative genes identified (RetNet, accessed 8 May 2012). However, none of these genes are major causative genes, with most causing less than 1% of cases. Currently, direct sequencing of all known arRP causative genes, exceeding 100 kilobase pair (kb) of coding sequence alone, is generally not a readily feasible option. Therefore, high-throughput technologies are vital for genetic characterization of arRP.

Pomares et al. recently developed a novel high-throughput technique that uses a combination of single nucleotide polymorphism (SNP) genotyping and cosegregation analysis to narrow the search for disease-causing genes in families affected by arRP, autosomal dominant RP (adRP), or LCA [14,15]. Non-discarded genes can then be further analyzed, such as by DNA sequencing. Additionally, all genes may be discarded in some cases, highlighting families in which to search for novel disease-causing genes.

The families with arRP studied by Pomares and colleagues [14,15] are mostly consanguineous (35/54 and 7/7 consanguineous families present in the 2007 and 2010 studies, respectively), allowing exclusion of genes by either lack of cosegregation with the disease or lack of homozygosity-by-descent. The current study aims to determine the efficiency of gene exclusion based on lack of cosegregation but not lack of homozygosity-by-descent in 31 non-consanguineous Australian families affected by arRP.

## Methods

### Subjects

All research participants for this study were sourced via the Australian Inherited Retinal Disease Register (AIRDR). Fifty-nine arRP affected individuals and 74 unaffected family members from 31 Australian families were selected, including 25 families with European Caucasian ancestry, three of Eastern Asian descent, two with Mediterranean ancestry, and one of Jewish descent. Informed consent was obtained from all participants in accordance with Sir Charles Gairdner Hospital Human Research Ethics Committee approval guidelines (Human Ethics Approval Number 2001–053).

### DNA collection and isolation

Either blood (30 ml EDTA) or saliva samples (2 ml; Oragene DNA Self-Collection kit OG-500; DNA Genotek Inc., Ottawa, Canada) were collected from each patient. DNA was extracted from buffy coat using the previously described salting out method [16-18] and from saliva samples using the manufacturer’s recommendations. Samples were stored at −40 °C in a cryofacility operated by the Western Australian DNA Bank.

### Single nucleotide polymorphism genotyping

Six SNPs for each of 28 genes known to cause arRP or the related disease, LCA, were identified for genotyping based on the work of Pomares and colleagues, who selected SNPs based on the informativity criteria according to dbSNP and SNPbrowser (2007), proximity to the gene, distribution throughout the gene and flanking regions, and presence in different haplotypic blocks [14,15]. These SNPs were used to develop a customized SNP genotyping assay on a SEQUENOM MassARRAY platform (San Diego, CA; Appendix 1). Four assays could not be optimized and were omitted from the study (rs1002098, lecithin retinol acyltransferase [*LRAT*]; rs717571, progressive rod-cone degeneration [*PRCD*]; rs10788333, retinal G protein coupled receptor [*RGR*]; and rs4982436, retinitis pigmentosa GTPase regulator interacting protein 1 [*RPGRIP1*]).

The following genes were analyzed: ATP-binding cassette, sub-family A (ABC1), member 4 (*ABCA4*), *AIPL1*, centrosomal protein 290 kDa (*CEP290*), ceramide kinase-like (*CERKL*), cyclic nucleotide gated channel alpha 1 (*CNGA1*), cyclic nucleotide gated channel beta 1 (*CNGB1*), crumbs homolog 1 (*CRB1*), cone-rod homeobox (*CRX*), guanylate cyclase 2D, membrane (retina-specific) (*GUCY2D*), Leber congenital amaurosis 5 (*LCA5*), *LRAT*, c-mer proto-oncogene tyrosine kinase (*MERTK*), nuclear receptor subfamily 2, group E, member 3 (*NR2E3*), neural retina leucine zipper (*NRL*), phosphodiesterase 6A, cGMP-specific, rod, alpha (*PDE6A*), phosphodiesterase 6B, cGMP-specific, rod, beta (*PDE6B*), *PRCD*, retinal degeneration 3 (*RD3*), retinol dehydrogenase 12 (all-trans/9-cis/11-cis) (*RDH12*), *RGR*, rhodopsin (*RHO*), retinaldehyde binding protein 1 (*RLBP1*), retinitis pigmentosa 1 (*RP1*), *RPE65*, *RPGRIP1*, S-antigen; retina and pineal gland (arrestin) (*SAG*), tubby like protein 1 (*TULP1*), and Usher syndrome 2A (autosomal recessive, mild) (*USH2A*). Each subject was genotyped, with all MassARRAY experiments performed using a 384-well Applied Biosystems’ GeneAmp 9700 thermocycler (Foster City, CA) at the Australian Genome Research Facility (Brisbane, Australia).

### Haplotyping and cosegregation analyses

Genotypes produced from the assays were used to determine haplotypes and perform cosegregation analyses. Each individual was manually haplotyped for five or six SNPs for each of the 28 genes. Genes were then excluded or included as possible disease-causing genes for each family depending on cosegregation of haplotypes with the disease. Genes were also included in cases of lack of informativeness.

### Statistical analyses

Linear regression was used to assess associations between family structure variables and the proportion of genes excluded out of the 28 genes analyzed. The number of affected and unaffected individuals analyzed, availability of maternal and paternal DNA, and total number of individuals in the sibship were analyzed univariately and multivariately. Variables that were significant at the 5% level following backwards elimination were retained in the final model. To estimate the potential costs of subsequent sequencing after SNP genotyping cosegregation analysis, we assumed a direct relationship between gene transcript size and genotyping costs (DNA Sequencing Costs). The data from this study were analyzed using the statistical package R (version 2.11.1) [19].

### Sequencing of candidate genes

**Figure 1:**
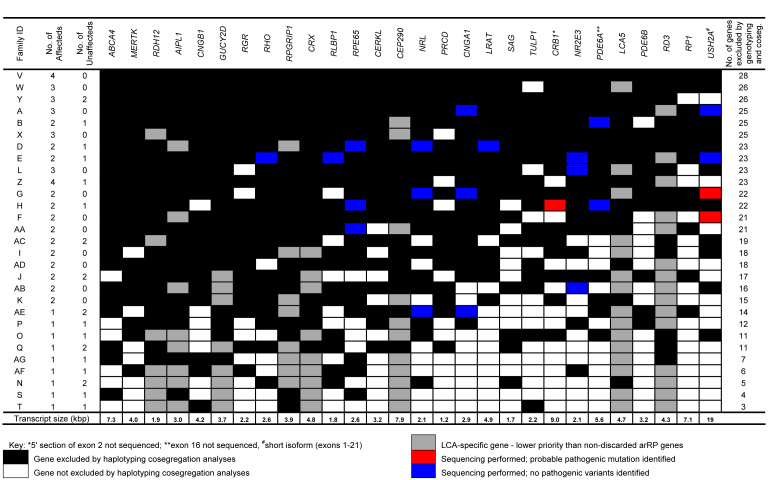
Gene exclusion results for each family. Summary of genes excluded from each family (highest to lowest number excluded) showing the number of affected and unaffected individuals analyzed, genes discarded with the SNP genotyping method (black) or sequencing analyses (blue), as well as non-discarded genes (white), LCA-specific genes (gray), and the number of genes excluded with SNP genotyping analyses. Likely mutations identified with DNA sequencing are shown in red.

Eleven candidate families most suitable for further investigation by direct sequencing and mutational screening of coding and flanking intronic regions of non-excluded genes were selected based on 1) the number of genes excluded by SNP genotyping cosegregation analyses, 2) the availability of published primers, and 3) the size of the non-excluded genes. Ten genes were then selected for sequencing analysis in these families, including *CNGA1*, *CRB1*, *LRAT*, *NR2E3*, *NRL*, *PDE6A, RHO*, *RLBP1*, *RPE65*, and *USH2A* (short isoform only; exons 1–21). DNA from all affected individuals was directly sequenced for up to four genes still potentially implicated following cosegregation analysis (Figure 1).

Primers were manufactured by Geneworks (Adelaide, Australia). Primer sequences (which were similar to previously published sequences [20-30]) and PCR conditions are available upon request. PCRs were undertaken using HotStarTaq Plus Master Mix (Qiagen, Hilden, Germany), and products were purified using the ExoSAP-IT method (USB Corporation, Cleveland, OH) according to the manufacturer’s instructions.

Samples were sequenced with dual direction sequencing on an ABI Prism 3730 48-capillary sequencer (Macrogen, Seoul, Korea) using the dideoxy nucleotide chain termination method [31]. Products were organized into contigs by amplicon and aligned with reference coding sequences in Sequencher 4.10.1 (Gene Codes Corporation, Ann Arbor, MI). Differences between the products and reference sequences were investigated using the NCBI SNP: GeneView database, the Human Gene Mutation Database (HGMD), and previously published work.

## Results

### Identification of false paternity

Following haplotyping analysis, false paternity was identified in two pedigrees due to inconsistencies between paternal alleles and haplotypes inherited by the children. In both families, 22 of the 28 genes analyzed displayed inconsistencies with identity by descent. However, both families had six genes that were haplotyped without any indication that the paternal DNA sample was not that of the biologic father. These two families were omitted from further analysis.

### Haplotyping and cosegregation analyses

Figure 1 displays a summary of the loci excluded for each pedigree following SNP genotyping cosegregation and sequencing analyses. In one family, all genes were discarded (Family V), and in some families, such as Family D, only LCA-specific genes remain (Figure 1).

**Figure 2:**
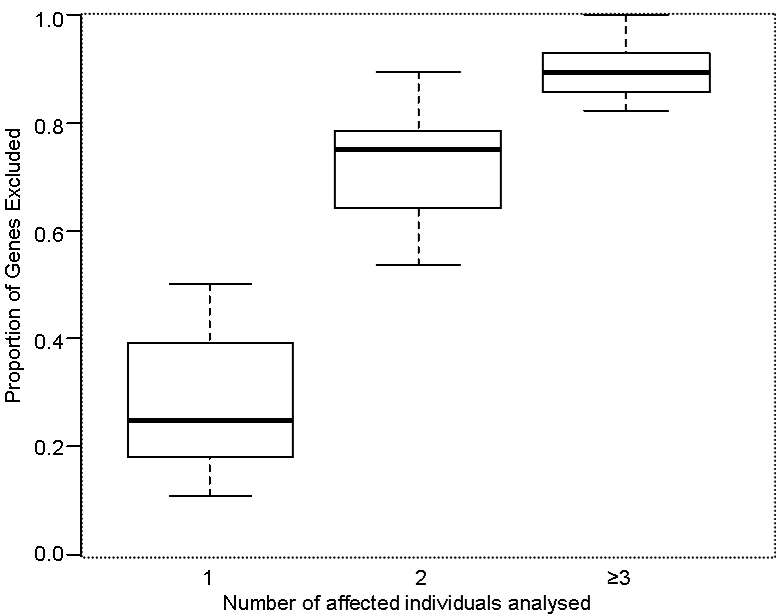
Proportion of genes excluded according to the number of affected individuals analyzed. Boxplot showing the proportion of genes excluded with the single nucleotide polymorphism genotyping cosegregation method, grouped according to the number of affected individuals analyzed. Shown on the boxplot are quartiles, the range of values for each group (whiskers), and the median values for each group (bold horizontal bars).

After haplotyping and cosegregation analyses were completed in the remaining 29 pedigrees, the mean number of candidate disease-causing genes excluded was 17.5 (standard deviation [SD]: 7.4; range: 3–28). Each gene was excluded from an average of 17 families, ranging from 11 to 23 (Figure 1). There was a marked difference in the number of genes that could be excluded in families based on the number of affected individuals analyzed (Figure 2), with a mean proportion of 0.290 of genes excluded for families with only one affected individual analyzed, compared to 0.898 excluded when three or more affected individuals were analyzed.

Linear regression revealed a significant association between the proportion of genes excluded and the affected individuals analyzed (p<0.0001), the unaffected individuals analyzed (p=0.0472), and the total number of individuals in sibship (p=0.0296). The parameter estimates, standard errors, and associated p values are displayed in Table 1. Associations were then tested multivariately and the number of affected individuals analyzed was found to be the only variable significantly associated with the proportion of genes excluded (p<0.0001).

**Table 1:** Influence of family structure variables on success of SNP genotyping cosegregation for exclusion of potentially causative loci

					**Linear regression**		
**Variable**	**Category**	**Number of families**	**Mean (SE) proportion of loci excluded**		**Parameter estimate (SE)**		**p-value**
Number of affected individuals analyzed*	1	9	0.290	(0.037)	−0.608	(0.056)	<0.0001
	2	13	0.714	(0.031)	−0.184	(0.053)	0.0017
	≥3	7	0.898	(0.042)	0	-	-
Number of unaffected individuals analyzed	0	12	0.768	(0.071)	0	-	-
	1	11	0.516	(0.074)	−0.252	(0.102)	0.0205
	2	6	0.548	(0.100)	−0.220	(0.122)	0.0830
Analysis of maternal DNA	No	5	0.743	(0.118)	0	-	-
	Yes	24	0.603	(0.054)	−0.140	(0.130)	0.2897
Analysis of paternal DNA	No	8	0.772	(0.089)	0	-	-
	Yes	21	0.571	(0.055)	−0.201	(0.105)	0.0667
Total number if individuals in sibship	2	4	0.384	(0.117)	−0.409	(0.158)	0.0156
	3	12	0.717	(0.068)	−0.076	(0.125)	0.5510
	4	8	0.509	(0.083)	−0.284	(0.134)	0.0441
	≥5	5	0.793	(0.105)	0	-	-

Overview of the influence of family structure variables on the mean number of genes excluded by SNP genotyping cosegregation analyses. *Remains significant following multivariate analysis. Abbreviations: SE, standard error; N, number; -, reference category.

### Mutation identification

Sequencing analyses of several non-discarded genes in 11 selected families resulted in likely disease-causing mutations being identified in three families (3/11=0.27). In each of these three families, a mutation was identified heterozygously in a non-discarded gene, but a second disease-causing mutation has yet to be identified.

In Family H, the known disease-causing variant *CRB1* c.2548 G>A (p.Gly850Ser) was identified heterozygously in both affected individuals. Likewise, a likely pathogenic *USH2A* variant, c.2276 G>T (p.Cys759Phe), was identified heterozygously in all affected individuals from Families F and G. The affected individuals from Families F and G all share one *USH2A* haplotype: CCTGCA (rs7519402, rs4253963, rs2669053, rs2034960, rs301760, and rs1544299). This haplotype was also identified in affected individuals from Families J and AF, for which *USH2A* was not excluded through intrafamilial cosegregation analysis.

The first 230 bp of *CRB1* exon 2 were not successfully sequenced, and therefore cannot be excluded from harboring a pathogenic mutation in Family H. Similarly, only the first 21 exons of *USH2A*, encoding the short isoform of the usherin protein, were directly sequenced.

## Discussion

In our cohort of non-consanguineous families, the SNP genotyping cosegregation method of gene exclusion successfully excluded approximately 90% of disease loci when at least three affected family members were genotyped (Figure 2). This success rate fell to below 30% in families for which only one affected individual was analyzed. The number of families in which each gene was excluded also varied, ranging from exclusion in 11–23 families (Figure 1). This result is likely to be due to the lack of informativity of some of the SNPs in the Australian population, with a higher frequency of inconclusive results in genes with the lowest exclusion rates, such as *PDE6B*, *RD3*, *RP1*, and *USH2A*. These exclusion rates may be improved by selecting the most informative SNPs for the population of interest.

Overall, our gene exclusion rate for non-consanguineous arRP families with more than one affected individual analyzed was similar to that reported by Pomares and colleagues [14]. Hence, in a non-consanguineous population this method appears to be effective only in a non-consanguineous population when used in families for which DNA samples are available from more than one affected individual, resulting in excluding the majority of candidate genes in these families. Subsequent sequencing analyses in 11 families resulted in the likely disease-causing gene being identified in three families. *CRB1* was one of six genes not discarded by SNP genotyping cosegregation analyses in Family H, with a heterozygous c.2548 G>A (p.Gly850Ser) mutation identified in *CRB1* in both affected individuals.

The c.2548 G>A change results in a missense amino acid mutation of a highly conserved glycine residue, which is completely conserved between the nine laminin A G-like domains of human CRB1, mouse Crb1, and *Drosophila* Crb [32]. The Gly850Ser mutation has been identified in individuals affected with RP [32,33], but not in 372 ethnically matched control individuals [32] or in 360 population controls [33]. This variant is predicted by PolyPhen-2 to be pathogenic, with the maximum score of 1 for the HumDiv and HumVar prediction models. Interestingly, a second disease-causing mutation has not yet been identified in Family H. A mutation may be present in exon 2 or the untranslated regulatory region or *CRB1*, which has not been fully sequenced.

A likely disease-causing mutation was also identified in Family F and Family G, with all affected individuals from both families heterozygous for the *USH2A* c.2276 G>T (p.Cys759Phe) variant. The cysteine residue at position 759 is highly conserved and is predicted to be crucial for protein structure through the formation of a disulfide bridge within the LE-motif of the usherin protein [34]. This variant is predicted by PolyPhen-2 to be “probably damaging,” with scores of 1 and 0.999 for the HumDiv and HumVar prediction models, respectively. The Cys759Phe variant is widely considered disease causing [34-42]; however, functional studies need to be performed to confirm this. *USH2A* is therefore considered the likely disease-causing gene in Family F and Family G, with a second disease-causing mutation still to be identified. Due to the extensive size of the *USH2A* gene, only exons 1–21 have been sequenced to date. A second disease-causing mutation could exist within exons 22–73 of the *USH2A* gene in these families. These exons may be further analyzed to search for a second disease-causing mutation.

Overall, the genetic findings from sequencing analyses in Families F, G, and H provide support for the SNP genotyping cosegregation method, but need to be confirmed by identifying a second disease-causing mutation in each case. Our results demonstrate that the SNP genotyping cosegregation technique could be readily implemented clinically for the genetic characterization of non-consanguineous populations. This technique will allow for rapid and relatively cheap determination of disease-causing loci in families affected by arRP and other genetically heterogeneous inherited diseases. Studies such as this may help identify individuals eligible for gene therapy trials, such as the *RPE65* human trials [7-9], as well as families in which to search for novel disease-causing genes.

The issue of misattributed paternity is pertinent to genetic studies such as this, in which results are often based on an analysis of parental DNA samples. Misattributed paternity rates have been reported to range from <1% to 30% in different populations [43-46]; however, the general non-paternity rate was estimated by Cerda-Flores and colleagues to be 11.8% [43]. In our study population, 6.5% (2/31) of the haplotyped families were found to have false paternity. Although this finding is consistent with previous studies, this issue of false paternity is not likely to alter the value of using this SNP genotyping cosegregation method to interrogate non-consanguineous populations. If, however, this method were used on only a small number of candidate genes, there would be a greater risk of misattributed paternity going undetected.

Since we commenced our study, several newly identified genes have been implicated in arRP, such as chromosome 2 open reading frame 71 (*C2ORF71*), chromosome 8 open reading frame 37 (*C8ORF37*), dehydrodolichyl diphosphate synthase (*DHDDS*), eyes shut homolog (Drosophila) (*EYS*), interphotoreceptor matrix proteoglycan 2 (*IMPG2*), male germ cell-associated kinase (*MAK*), retinol binding protein 3, interstitial (*RBP3*), tetratricopeptide repeat domain 8 (*TTC8*), and zinc finger protein 513 (*ZNF513*; RetNet; accessed 8 May 2012). To keep this method up-to-date and clinically applicable, these genes need to be incorporated into the SNP genotyping pipeline. The current cost of the SNP genotyping method is about US$150 per person to genotype a cohort of 100 individuals using the method described herein. To incorporate 5–10 newly identified genes by the set-up of at least two additional multiplexes, the cost would be about US$175 per person. Naturally, the ultimate cost saved by excluding loci using this cosegregation technique depends on the size of the candidate gene excluded (Figure 1). Nonetheless, we estimate that excluding more than 89%, 70%, or 30% of the potential loci studied here would result in an approximate seven-, 4.8-, or threefold savings in direct genotyping costs, respectively.

In families in which all genes are discarded by SNP genotyping cosegregation analyses, such as Family V in the current study, whole exome or whole genome analyses can be pursued to identify novel disease-causing genes. A next-generation sequencing approach should also be considered for families such as Families A, D, and E, which have only LCA-specific genes remaining and are unlikely to be affected with LCA based on age of onset and other phenotypic information.

These next-generation methods are becoming essential for identifying new disease-causing genes, but remain expensive for analyzing and excluding a small number of candidate loci, even as costs fall below that set for the elusive “thousand dollar genome.” Thus, the SNP genotyping method is currently economically competitive; however, the cost of keeping this method up-to-date must be considered. Alternative approaches for genetic characterization may eventually be more cost-effective, especially with progress in whole-genome screening and the likely escalation in discovery of novel disease-causing genes. Nonetheless, as the rate of identifying disease-associated loci slows, keeping a disease-specific genotyping array updated will be less of a burden.

In summary, the SNP genotyping cosegregation method of gene exclusion is a useful means for rapidly screening and excluding candidate loci in a non-consanguineous population. Improved methods for genetic characterization of RP and related diseases are vital for identifying disease-causing genes and the subsequent application of gene therapies. The rapid progression of technology and identification of disease-causing genes is assisting in developing potential therapies and providing encouraging prospects for studying and treating this debilitating retinal disease.

## Appendix 1. Primers.

Primers used by Australian Genome Research Facility (Brisbane, Australia) for genotyping five or six single nucleotide polymorphisms (SNPs; rs accession numbers shown) for 28 genes known to cause autosomal recessive retinitis pigmentosa and/or Leber congenital amaurosis. Both forward and reverse primers have a 5′ 10mer tag to alter the mass and is non-binding to the template DNA. Some iPlex extension primers contain 5′ tags to alter the mass of the probe, which are non-binding to the template DNA and are represented by lower case nucleotides. Abbreviations: Tm, primer melting temperature. To access the data, click or select the words “Appendix 1.” This will initiate the download of a compressed (pdf) archive that contains the file.
